# Neuroprotective Potential of Gentongping in Rat Model of Cervical Spondylotic Radiculopathy Targeting PPAR-*γ* Pathway

**DOI:** 10.1155/2017/9152960

**Published:** 2017-11-05

**Authors:** Wen Sun, Kang Zheng, Bin Liu, Danping Fan, Hui Luo, Xiaoyuan Qu, Li Li, Xiaojuan He, Jianfeng Yi, Cheng Lu

**Affiliations:** ^1^College of Chemical and Biological Engineering, Yichun University, Yichun 336000, China; ^2^Institute of Basic Research in Clinical Medicine, China Academy of Chinese Medical Sciences, Beijing 100700, China; ^3^School of Life Science and Engineering, Southwest Jiaotong University, Chengdu 610000, China; ^4^Beijing Yjheal Medical Research Center, Beijing 100100, China

## Abstract

Cervical spondylotic radiculopathy (CSR) is the most general form of spinal degenerative disease and is characterized by pain and numbness of the neck and arm. Gentongping (GTP) granule, as a classical Chinese patent medicine, has been widely used in curing CSR, whereas the underlying mechanism remains unclear. Therefore, the aim of this study is to explore the pharmacological mechanisms of GTP on CSR. The rat model of CSR was induced by spinal cord injury (SCI). Our results showed that GTP could significantly alleviate spontaneous pain as well as ameliorate gait. The HE staining and Western blot results showed that GTP could increase the quantity of motoneuron and enhance the activation of peroxisome proliferator-activated receptor gamma (PPAR-*γ*) in the spinal cord tissues. Meanwhile, immunofluorescence staining analysis indicated that GTP could reduce the expression of TNF-*α* in the spinal cord tissues. Furthermore, the protein level of Bax was decreased whereas the protein levels of Bcl-2 and NF200 were increased after the GTP treatment. These findings demonstrated that GTP might modulate the PPAR-*γ* pathway by inhibiting the inflammatory response and apoptosis as well as by protecting the cytoskeletal integrity of the spinal cord, ultimately play a neuroprotective role in CSR.

## 1. Introduction

Cervical spondylotic radiculopathy (CSR) is one of the most general form of spinal degenerative disease [1]. The clinical manifestations of CSR focus on pain and numbness of the neck and arm as well as restricted neck movement, which greatly impact the patient's life and work [2]. The orthopedic of TCM theory holds that both the static system and the dynamic system are critical in maintaining normal position and function of the cervical spine. The imbalance of both static and dynamic forces can result in a degeneration of posterior column stability and then lead to rapid degeneration of the cervical intervertebral discs, thus causing a series of syndromes distributed along the spinal nerve roots [3, 4]. However, poor understanding of the pathobiology of CSR has limited the therapeutic progression of neurological dysfunction [5]. The strategies currently approved for treatment of CSR include operative treatment and nonoperative treatment categories (including drugs, traction, physical therapy, functional exercise, etc.) [6, 7], in which Chinese herbal medicine therapy has occupied an important position [8–10].

*Gentongping* (GTP) granule is a classic traditional Chinese medicine (TCM) granule refined from several Chinese herbal medicines, such as Radix Paeoniae Alba, Radix Puerariae, *Carthamus tinctorius*, and so forth. It is widely produced in China in accordance with the China Pharmacopoeia standard of quality control and generally used for the treatment of cervical and lumbar spine disease [11, 12]. In preparation technology studies, Zhu et al. obtained a high-efficiency formulation [13]. High-performance liquid chromatography (HPLC) was also repeatedly used in the determination of principal component analysis of GTP [14, 15]. For pharmacodynamic evaluation, Chen et al. and Peng et al. observed the therapeutic efficacy in improving the symptoms and signs of patients with cervical spondylosis radiculopathy [16, 17]. Although the herb product has been applied generally, the scientific basis of GTP treatment on CSR remains unclear.

Network pharmacology, a novel research field that elaborates the potential mechanisms of biological systems by analyzing diverse biological networks, may have the capacities to address the relationship between complex Chinese herbal medicine and disease [18]. According to our previous studies [19–21], we have obtained structural information of the composite compounds of each ingredient in GTP from TCM Database@Taiwan [22] (http://tcm.cmu.edu.tw/), which is currently the largest noncommercial TCM database worldwide. In total, we collected the structural information of 15 compounds for Raidix Paeoniae Alba, 22 compounds for Radix Puerariae, and 23 compounds for *Carthamus tinctorius*. We found that Galuteolin was the main component of Raidix Paeoniae Alba and arachidic acid was the main component of Radix Puerariae and *Carthamus tinctorius*. According to the PubChem database [23] (https://pubchem.ncbi.nlm.nih.gov/), we searched for an important protein peroxisome proliferator-activated receptor gamma (PPAR-*γ*), which was the target protein of arachidic acid (Supplementary Table 1 available online at https://doi.org/10.1155/2017/9152960). PPAR-*γ* and its downstream molecules are involved in nociception [24]. PPAR-*γ* coactivator, a cluster of nuclear transcriptional coactivators, plays an important role in several metabolism procedures including mitochondrial biogenesis, thermogenesis, respiration, insulin secretion, and gluconeogenesis, as well as the regulation of inflammation [25, 26]. The PPAR-*γ* agonists have potential therapeutic actions on nervous disorders, including neuropathic pain, and have been regarded as promising therapeutic strategies for spinal cord injury patients [27, 28]. Thus, we speculated that the PPAR-*γ* pathway might be involved in the GTP treatment of CSR for effective neuropathic pain relief.

Spinal cord injury (SCI) results in a gradually amplified necrotic area of cavitation, due to secondary neuronal death accelerated by acute inflammation, edema, apoptosis, and glial scarring [29–31]. Previous studies have shown that the rat model of SCI is a clinically relevant model [32]. Therefore, the purpose of this study was to implement the SCI model to investigate the basic therapeutic mechanism of GTP on CSR concerning the PPAR-*γ* pathway.

## 2. Materials and Methods

### 2.1. Animals

Forty male Sprague-Dawley (SD) rats with mean weights of 190–210 g were obtained from Beijing Vital River Laboratory Animal Technology Co. Ltd. (China). The animals were maintained in a specific pathogen-free environment at an appropriate temperature and humidity and were allowed free access to standard rodent chow and water. Body weight was recorded once every three days. Before surgery, the rats were kept in the cages for 7 days for adaptation to the environment and the exercise [33]. The rodent license of the laboratory (number SYXK-2010-0032) was issued by the National Science and Technology Ministry of China. Animal care and experimental protocols complied with the Research Ethics Committee of 163 Institute of Basic Theory of Chinese Medicine, China Academy of Chinese Medical Sciences.

### 2.2. Spinal Cord Compression Model

Male SD rats were anesthetized with intraperitoneal injection of 10% chloral hydrate (0.30 mL/100 g). Then, the surgical area was shaved and disinfected with 75% ethanol and betadine [5]. A 3.5 cm midline incision was incised at the C4-T1 area in nuchal, and the skin and superficial muscles were retracted, followed by the exposure of the C4-C6 laminas. The cervical laminas were identified by counting from T1. After that, ligamentum flavumin of C5-C6 and C6-C7 were resected, the periosteum of C6 lamina was removed, and then a nylon suture measuring 15 mm long with a 0.5 mm diameter was implanted underneath the C6 lamina. The dura mater underneath was carefully separated from the lamina to avoid a tear and cerebral spinal fluid (CSF) leak. In the animals assigned to the sham operation group, the implantation passed through the dorsal epidural space [34]. Multilayer tissue closure was then performed [5]. After recovery on a heating pad, the rats were housed in individual cages and given food and water ad libitum.

### 2.3. Drug Preparation and Administration

After one week of SCI operation, all rats received drug administration. The drug concentrations and administration profiles were implemented based on the specification and kinetic properties. The treatment group was administered with GTP (Beijing Handian Pharmaceutical Co. Ltd., China) at a concentration of 0.32 g/100 g dissolved in saline solution. The positive control group was administered with fenbid (Glaxo Smith Kline Investment Co. Ltd., China) at a concentration of 4 mg/100 g dissolved in saline solution. The normal group, sham group, and model group were all given with saline with the same volume [35]. All rats were administered once a day for 5 weeks before sacrificed.

### 2.4. Neurobehavioral Assessments

Animals received a neurobehavioral test on the seventh day after the SCI operation. The rats were placed in an observation box. The duration of left forepaw leaving from the bottom was recorded over 15 min divided by 3 times, and the degree of spontaneous pain was evaluated by the duration. Meanwhile, the postures of rest and walk were monitored. The concrete process for evaluating gait was executed according to the previous study performed by Kawakami et al. and Dubuisson and Dennis [36, 37], and the rating scale was recorded as follows: 1 = normal gait without forepaw deformity; 2 = normal gait with a marked forepaw deformity such as flexed and/or inverted paw or slight gait instability with a paw drop when walking; and 3 = severe gait instability with motor paresis of the ipsilateral left forepaw. The assessment was implemented once a week for a period of 6 weeks.

### 2.5. Electrophysiology

At 6 weeks postsurgery, sensory-evoked potentials (SEPs) were recorded in each group. The rats were immobilized on a stereotaxic board in a prone position. After removing interlaminar ligaments between C5 and C6, two pairs of steel needle electrodes were positioned, respectively, at C6 ganglion and Erb's point for recording evoked potentials. Another pair of steel needle electrodes was inserted into the median nerve of the left forepaw as stimulating electrodes. A constant current stimulus of 0.1 ms in duration and 3.5 mA in intensity was applied at a rate of 2 Hz. At a bandwidth of 10–3000 Hz, a total of 800 SEPs were averaged and replicated. The evoked potential amplitudes were measured as the voltage difference from the peak of the first positive peak (P1) to the peak of the first negative peak (N1) [5].

### 2.6. Histopathology

The specimens were immersed into 4% paraformaldehyde in 1x phosphate-buffered saline (4% PFA) and stored at 4°C for fixation. After being fixed for 48 h, they were removed and placed in fresh fixative. Fixed tissue samples were disposed of paraffin embedding and dehydration routinely. A total of 5 serial cross sections with thickness of 5 *μ*m were obtained from each rat and processed with hematoxylin and eosin (HE) staining. The images were obtained using a light microscope (Carl Zeiss, Germany). Motoneurons were identified by the presence of large nuclei and Nissl bodies, which densely stained in the cytoplasm [38]. All sections were evaluated morphologically by the same pathologist who was blinded to each group.

### 2.7. Western Blot Analysis

Cytosolic extracts were prepared as described previously [39], with slight modifications. Briefly, 2 cm tissue segments containing the lesion from each rat were processed. The level of PPAR-*γ*, TNF-*α*, Bax, Bcl-2, and NF200 was quantified in the cytosolic fraction from spinal cord tissue. Protein concentration was determined with the assay kit (Biotime Biotechnology, China). Proteins were separated electrophoretically and transferred to nitrocellulose membranes. Membranes were blocked with 5% (*w*/*v*) nonfat dried milk in buffered saline for 45 minutes at room temperature and subsequently probed with the following specific antibodies: anti-PPAR-*γ* (1 : 1000, *v*/*v*, Abcam, UK), anti-TNF-*α* (1 : 1000, *v*/*v*, Abcam, UK), anti-Bcl-2 (1 : 500, *v*/*v*, Abcam, UK), anti-Bax (1 : 2000, *v*/*v*, Abcam, UK), and anti-NF200 (1 : 500, *v*/*v*, Abcam, UK) in 1 × TBST, 5% *w*/*v* bovine serum albumin (BSA) at 4°C overnight. Membranes were incubated with peroxidase-conjugated goat anti-rabbit IgG (1 : 2000, *v*/*v*, ZSGB Bio. Co. Ltd., Beijing, China) secondary antibody for 2 h at room temperature [40].

To ascertain if Western blots were loaded with equal amounts of protein lysates, they were also incubated in the presence of the antibody anti-GAPDH protein (1 : 1000, *v*/*v*, Abcam, UK). Signals were detected with the chemiluminescence detection system (Thermo Fisher Scientific Inc., USA), according to the manufacturer's instructions [40]. Gel-Pro Plus Analyzer software (Media Cybernetics) was used for integrated optical density (OD) analysis [35].

### 2.8. Immunofluorescence

Tissue samples were deparaffinized and rehydrated, and after boiling in 0.1 M citrate buffer for 1 min, detection of TNF-*α* and GFAP was carried out. In order to minimize nonspecific adsorption, the sections were incubated in 1% (*v*/*v*) bovine serum albumin in PBS for 25 min and then incubated with polyclonal rabbit anti-TNF-*α* (1 : 100, *v*/*v*, Abcam, UK) and goat monoclonal anti-GFAP (1 : 1000; *v*/*v*, Abcam, UK) antibody in a humidity cabinet overnight at 4°C. Using PBS, the sections were washed three times for 15 min, followed by incubating with anti-rabbit Alexa Fluor-488 antibody (1 : 200, *v*/*v*, Santa Cruz, USA) and anti-goat Alexa Fluor-594 antibody (1 : 200, *v*/*v*, Santa Cruz, USA) secondary antibody for 2 h at room temperature. For nuclear staining, 1 *μ*g/mL DAPI in PBS was added and then rinsed for three times again. Fluorescence quenching agent was added to maintain fluorescence sensitization. All images were captured at a magnification of 200x on a fluorescence microscope (Carl Zeiss, Germany) [40].

### 2.9. TUNEL Staining

To determine the ability of GTP treatment on preventing apoptotic cell death after SCI, terminal deoxynucleotidyl transferase-mediated dUTP nick end labeling (TUNEL) was performed with the in situ cell death detection kit, POD (Roche Applied Science, USA). In order to block endogenous peroxidase activity, the sections were immersed in 3% H_2_O_2_ for 15 min in the dark. Sections were permeated with proteinase K solution (20 *μ*g/mL in 10 mM Tris/HCl, pH 7.5) at 37°C for 15 min and then rinsed in phosphate-buffered saline (PBS) for three times, followed by application of TUNEL reaction mixture in a humidified chamber at 37°C for 1 h. To label apoptotic cells, the sections were then incubated for 30 min at 37°C with converter-POD. The sections were rinsed in PBS, treated with DAB substrate solution, and washed again with PBS. The negative control was incubated in Label Solution without terminal transferase, and as a positive control, DNase I recombinant was added 10 min prior to labeling procedures to induce DNA strand breaks. Each section was imaged under a light microscope (Carl Zeiss, Germany), and the quantity of TUNEL positive cells was counted [35].

### 2.10. Enzyme-Linked Immunosorbent Assay (ELISA)

The level of TNF-*α* in the serum of rats was measured by ELISA using a commercial ELISA kit (Abcam, Germany) according to the manufacturer's instructions [41] (Supplementary Figure 1).

### 2.11. Statistical Analysis

All data are represented as the mean ± SD. Statistical differences were analyzed by Student's *t*-test for paired data with GraphPad Prism 7 software (USA). *P* < 0.05 was considered as statistically significant.

## 3. Results

### 3.1. Effects of GTP on Locomotor Recovery and Weight in Rats

**Figure 1:**
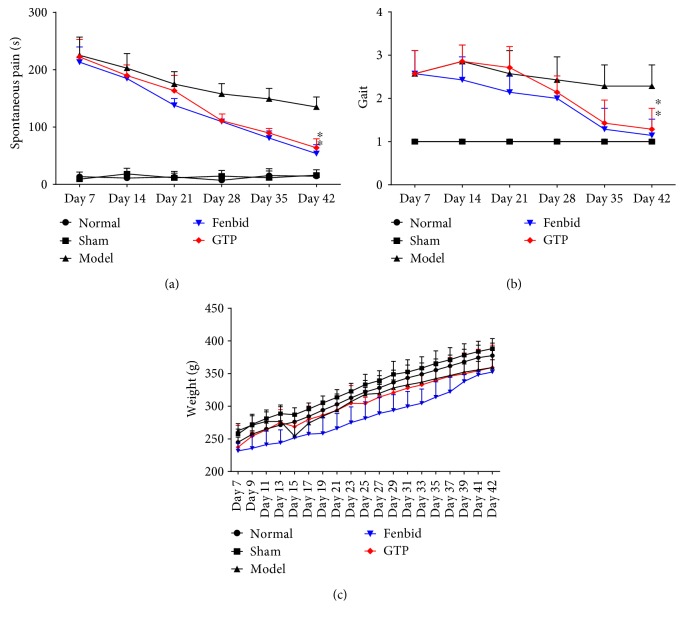
Effects of GTP on locomotor recovery and weight in rats. (a) Spontaneous pain. (b) Gait. (c) Weight. Compared to the model group, GTP treatment significantly reduced the duration of spontaneous pain (*P* < 0.05). The data are expressed as the mean ± SD, *n* = 8. ^∗^*P* < 0.05 versus model group.

The scores of spontaneous pain and gait were assessed by Kawakami et al. and Dubuisson and Dennis. In the last week, the scores of spontaneous pain showed a significant decrease in the GTP group compared to the model group (*P* < 0.05, Figure 1(a)); meanwhile, the gait score of the GTP group showed an obvious decrease compared to the model group (*P* < 0.05, Figure 1(b)). The body weights of all groups were not significantly different.

### 3.2. Effect of GTP on Somatosensory-Evoked Potentials (SEPs) in Rats

**Figure 2:**
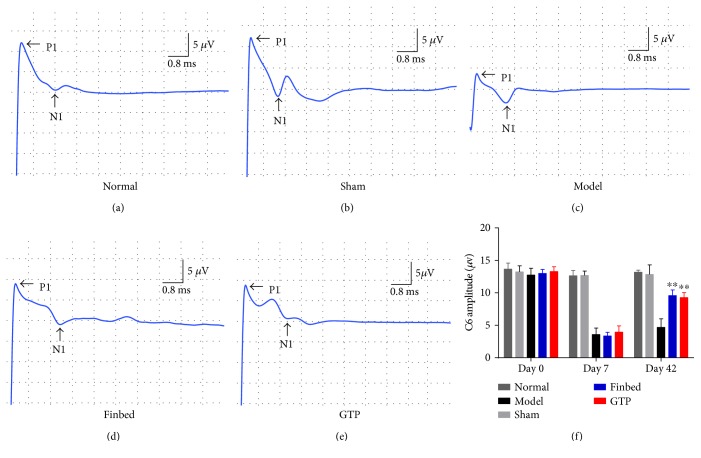
Effects of GTP on somatosensory-evoked potentials (SEPs) after SCI in rats. (a, b, c, d, e) The electrophysiological detection at C6 ganglion. The evoked potential amplitudes were measured as the voltage difference from the peak of the first positive peak (P1) to the peak of the negative peak (N1). (f) The GTP group had significantly higher peak amplitude than the model group (*P* < 0.01). The data are expressed as the mean ± SD, *n* = 8. ^∗∗^*P* < 0.01 versus model group.

Changes of axonal function were examined using electrophysiology detection [35]. SEP amplitude was significantly higher in the GTP group than in the model group (*P* < 0.01, Figure 2(f)). Peak latencies were not significantly different among groups.

### 3.3. Effect of GTP on Histological Alterations of the Spinal Cord Tissues

**Figure 3:**
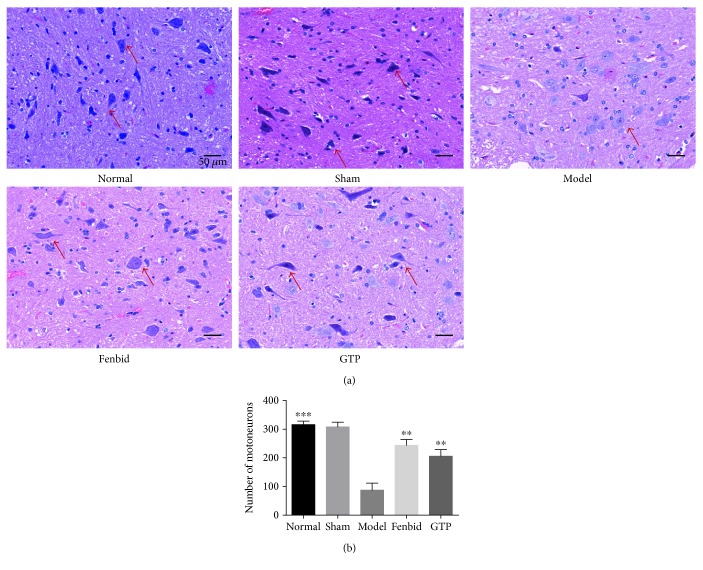
Effect of GTP on histological alterations of the spinal cord tissues. (a) HE staining (200x) of the spinal cord tissues. In the normal group, the motoneuron nuclei were densely blue stained, while in the model group, the motoneuron nuclei were stained light blue. (b) In the model group, the number of motoneurons was dramatically decreased compared to the normal group (*P* < 0.001). In GTP group, the number of motoneurons was significantly increased compared to the model group (*P* < 0.01). The data are expressed as the mean ± SD, *n* = 8. ^∗∗^*P* < 0.01 and ^∗∗∗^*P* < 0.001 versus model group.

HE staining was applied to analyze histopathological alterations. The number of motoneurons in the chronic compression site reduced progressively. In the normal group, a portion of motoneurons were observed with condensed nuclei and darkly stained cytoplasm, and no histopathological changes were observed (Figure 3(a)). In the model group, motoneurons appeared as irregular morphologies and the number of motoneurons was significantly decreased compared to the normal group (*P* < 0.01, Figure 3(b)). The treatment of GTP markedly improved the pathological conditions.

### 3.4. Effects of GTP on PPAR-*γ* Expression in Spinal Cord Tissues

**Figure 4:**
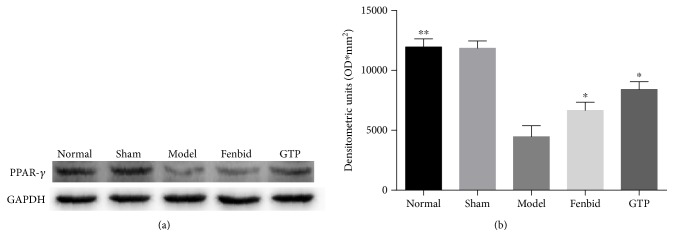
Effects of GTP on PPAR-*γ* expression in spinal cord tissues. The detection of PPAR-*γ* level in spinal cord tissues was assayed by Western blot analysis. The level of PPAR-*γ* in the model group was markedly lower compared to the normal group (*P* < 0.01). The level of PPAR-*γ* was obviously increased in the GTP group (*P* < 0.05). The data are expressed as the mean ± SD, *n* = 8. ^∗^*P* < 0.05 and ^∗∗^*P* < 0.01 versus model group.

To determine whether the expression of PPAR-*γ* is associated with the protective effects of GTP against SCI-induced neurotoxicity, the protein levels of PPAR-*γ* were determined by Western blot analysis (Figure 4(a)). The level of PPAR-*γ* in the model group was markedly lower compared to the normal group (*P* < 0.01, Figure 4(b)), and the expression of PPAR-*γ* was obviously increased when the rats were treated with GTP (*P* < 0.05, Figure 4(b)).

### 3.5. Effects of GTP on TNF-*α* Expression in Spinal Cord Tissues

**Figure 5:**
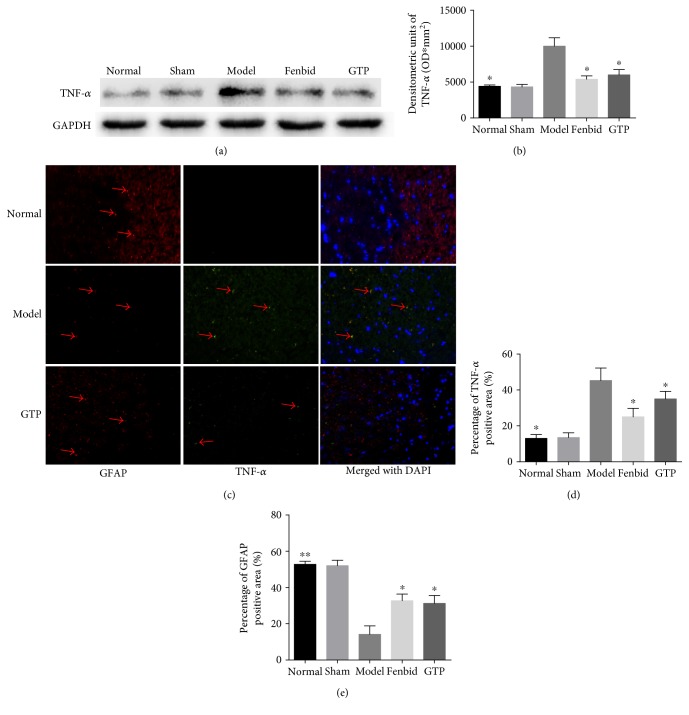
Effects of GTP on TNF-*α* expression in spinal cord tissues. (a, b) The detection of TNF-*α* level in spinal cord tissues was assayed by Western blot analysis. (c) Representative photomicrographs showing immunofluorescence staining of TNF-*α* (green), GFAP (red), and merging of the photographs (200x). (d, e) Statistical analysis of the TNF-*α*- and GFAP-positive staining areas. In the model group, the expression of TNF-*α* was obviously increased (*P* < 0.05) and GFAP was decreased compared to the normal group (*P* < 0.01). In the GTP group, the expression of TNF-*α* was obviously decreased (*P* < 0.05) and GFAP was increased compared with the model group (*P* < 0.05). The data are expressed as the mean ± SD, *n* = 8. ^∗^*P* < 0.05 and ^∗∗^*P* < 0.01 versus model group.

The protein content of TNF-*α* in the spinal cord of the rats was detected by Western blot. The rats in the model group had a higher TNF-*α* protein level compared with the rats in the normal group (*P* < 0.05, Figures 5(a) and 5(b)). By contrast, treatment with GTP significantly reduced the TNF-*α* protein level in contrast with SCI alone (*P* < 0.05, Figures 5(a) and 5(b)). Our data thus indicate that GTP inhibits TNF-*α* protein expression in the injured spinal cord.

Moreover, in order to confirm astrogliosis and TNF-*α* expression and to localize TNF-*α* to certain cell types, we implemented immunofluorescence staining. Spinal cord tissues were double stained with antibodies against TNF-*α* (green) and GFAP (red) (Figure 5(c)). The results revealed increased astrogliosis (GFAP^+^ cells) in SCI rat such as activation of the microglia. The expression of TNF-*α* protein was notably increased in SCI-treated rats (*P* < 0.05, Figure 5(d)), while GFAP immunoreactivity and microglia activation were reduced (*P* < 0.05, Figure 5(e)). The yellow spots represented the colocalization between GFAP and TNF-*α*.

### 3.6. Effects of GTP on Intrinsic Apoptosis

**Figure 6:**
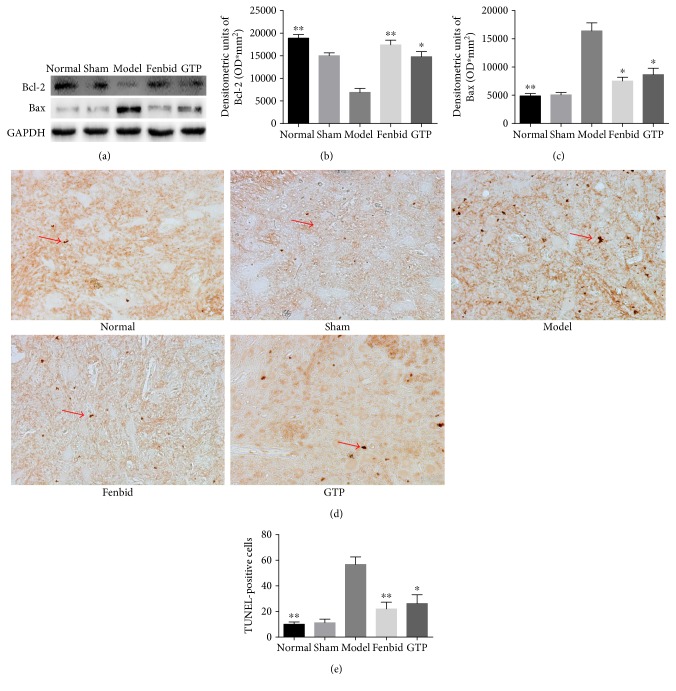
Effects of GTP on intrinsic apoptosis. (a) The detection of Bcl-2 and Bax levels in spinal cord tissues with Western blot analysis. In the model group, the expression of Bax was significantly increased (*P* < 0.01) and Bcl-2 was decreased compared with the normal group (*P* < 0.01). In the GTP group, the expression of Bax was obviously decreased (*P* < 0.05) and Bcl-2 was increased compared with the model group (*P* < 0.05). (d) TUNEL (200x) staining of the spinal cord tissues. (e) In the GTP group, the number of TUNEL-positive cells was obviously decreased compared to the model group. The data are expressed as the mean ± SD, *n* = 8. ^∗^*P* < 0.05 and ^∗∗^*P* < 0.01 versus model group.

The detection of Bcl-2 and Bax levels in spinal cord tissues with Western blot analysis (Figure 6(a)). The expression of Bcl-2 in the GTP group was obviously higher than in the model group (Figure 6(b)). A substantial increase in Bax expression was also found in spinal cord tissues from SCI; GTP treatment significantly attenuated Bax level in the spinal cord (Figure 6(c)).

Apoptotic cells emerged throughout the white and gray matter of the spinal cord. SCI-induced apoptosis in the spinal cord was detected by TUNEL staining. In the normal group, there was almost no TUNEL-positive cell, and the neurons presented with a normal morphology (Figure 6(d)). In the model group, the number of TUNEL-positive cells was significantly increased when compared to the normal group (*P* < 0.01, Figure 6(e)). However, GTP treatment markedly reduced the number of TUNEL-positive cells in contrast with the model group (*P* < 0.05, Figure 6(e)).

### 3.7. Effect of GTP on Cytoskeletal Integrity of Axons

**Figure 7:**
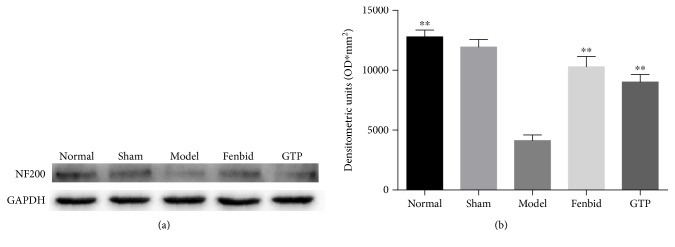
Effect of GTP on the cytoskeletal integrity of axons. (a) The detection of NF200 level in spinal cord tissues was assayed by Western blot analysis. (b) In the model group, the expression of NF200 was significantly decreased compared with the normal group (*P* < 0.01). In the GTP group, the expression of NF200 was significantly increased compared with the model group (*P* < 0.01). The data are expressed as the mean ± SD, *n* = 8. ^∗∗^*P* < 0.01 versus model group.

In this study, the cytoskeletal integrity of the cervical (C6) spinal cord was assessed. Homogenates of 8 mm of the spinal cord around the lesion epicenter were utilized for Western blot analysis to detect the cytoskeletal protein neurofilament 200 (NF200). It has been elaborated that after SCI, calcium-mediated excitotoxicity leads to the attenuation of NF200 [42]. Compared with the normal group, the expression of NF200 in the GTP treatment group was mildly lower, but far higher than in the model group (*P* < 0.05, Figure 7).

## 4. Discussion

Cervical spondylotic radiculopathy is one of the most common type of cervical degenerative disease. In recent years, many studies have confirmed that nonoperative therapy has more evident effects than operative therapy on the optimized scheme of CSR [6, 7]. Spinal cord injury, as the most common cause of CSR, has a high prevalence and a profound impact on patients and on society as a whole [40]. In the current study, a compression material was used to result in a progressive injury in rats, which then produced neurological deficits and ultimately formed a clinically relevant model [5]. This model is critical for screening neuroprotective agent and determining their mechanisms for the development of therapy.

*Gentongping* granule is one of the classical herbal products for treating CSR [16]. Many long-term clinical trials have reported that this medicine exerts admirable neuroprotective effects on CSR patients [11, 16, 17]. However, few pharmacological studies have been carried out on the specific mechanism. This study demonstrated that GTP improved behavioral function and somatosensory-evoked potentials, such as amelioration of gait and amplitude. Behavioral assessment is a prevalent method for accessing the degree of neural injury [36, 37]. In the present study, compared with the normal group, the rank of spontaneous pain and gait exhibited an obvious deterioration in the model group. Moreover, behavioral scores were significantly different between the GTP group and the model group. Based on the powerful spinal cord self-healing action, the behavioral function of rodents following SCI gradually recovered in the late stage of the experiment. However, the animal models in this study were sacrificed once behavioral assessment demonstrated a significant difference between the GTP group and the model group, and the effect of the self-healing could be ignored. Therefore, in the present study, GTP restored the behavioral function, indicating a satisfactory improvement on neuropathic pain.

Hematoxylin and eosin staining was used to determine whether spinal cord neurons underwent pathological changes. In normal neuronal nuclei, there are numerous Nissl bodies that are associated with the nutritional condition of neurons, and the quantity of Nissl bodies are often used to indicate the neural state [43]. The smaller the quantity is, the more severe the injury becomes. In extremely serious pathological conditions, few Nissl bodies could even been observed [44]. In the present study, spinal cord tissues stained with HE exhibited densely dark blue nuclei in the normal group, while in the model group, it exhibited a light blue staining in nuclei, which meant that the Nissl bodies which existed in neuronal nuclei were shrunken. For the GTP group, we found that the color was deepened, which demonstrated a restored neuronal number in the spinal cord, indicating that neuronal function was retained.

Peroxisome proliferator-activated receptor gamma, one of PPAR superfamily members, has been observed in multiple immunocytes, such as monocytes, macrophages, and lymphocytes [45]. In this regard, we performed a Western blot analysis to determine the PPAR-*γ* level in the spinal cord. We found that the expression of PPAR-*γ* markedly decreased in the model group, whereas in the GTP group, the expression was significantly increased. In recent years, many researchers have indicated that PPAR-*γ*-mediated mechanisms directly or indirectly favor the neuroprotective events [46]. The neuroprotection could also be abolished by a PPAR-*γ* antagonist [47]. Furthermore, growing evidence confirmed that there are intimate associations between PPAR-*γ* and SCI in neuroprotection [29, 31]. Using database retrieval, we also found that PPAR-*γ* was a common target protein of Radix Puerariae and *Carthamus tinctorius*, which are two critical components of GTP. The results indicated that GTP might exert its therapeutic action on PPAR-*γ*.

Recent studies suggest that neuroimmune activation involving the activation of neurons and the releasing of inflammatory mediators contributes to neuropathic pain [48]. The expression of proinflammatory cytokines, such as TNF-*α*, has been well demonstrated in regulating the precise cellular events including activating astrocytes in SCI [40]. PPAR-*γ*, being highly expressed in immunocytes, such as monocytes, macrophages, and lymphocytes [45], holds a pivotal role in adjusting inflammatory responses by regulating the activity of TNF-*α* in neuroprotective events [49, 50]. Furthermore, PPAR-*γ* agonists curtail the inflammatory cytokines expressed in SCI models, indicating that the prevention of inflammation is a contributing factor to neuroprotection [31]. This study exhibited that TNF-*α* level in the model group was substantially augmented compared with the GTP group. In immunofluorescence staining, we applied GFAP, a specific marker of astrocyte, to confirm the specific cell type in which TNF-*α* existed. The images further determined that the inflammatory cytokine was activated exactly in neurons. As a consequence, the evidences suggested a therapeutic potential in neuroinflammatory disorders that might be attributed to a PPAR-*γ*-involved mechanism. Recently, reduction of GFAP has been reported in major neuropathic disease [51]. Evidence showed that the inhibition of the astrocyte reaction and astrogliosis may be linked to the reduction of neuronal damage [52], which is considered as a physical barrier for neuron regeneration. Moreover, in our future studies, consideration of the expression of GFAP could be very appreciable.

Additionally, several researchers have suggested that the neuronal injury induced by SCI may be intimately associated with the activation of apoptotic proteins, such as antiapoptotic protein B-cell lymphoma 2(Bcl-2) and proapoptotic protein Bcl-2-associated X (Bax) [53]. In our study, GTP was shown to have an antiapoptotic effect via increasing Bcl-2 and decreasing Bax expression in the spinal cord. In accordance, the rodents in the model group were shown to have increased Bax expression and decreased Bcl-2 expression in the spinal cord, while in the GTP group, the expressions of the two apoptotic proteins were reversed, which demonstrated that the apoptotic case was attenuated after GTP administration. This outcome was further verified by the TUNEL assay, presented as a large quantity of TUNEL-positive cells in the model group, while the number significantly decreased in the GTP group. As a previous study exposed, PPAR-*γ* agonists contribute to the expression of Bcl-2 in various neurons and might facilitate cellular survival. In addition, the contribution was prevented by PPAR-*γ* antagonist [53]. Based on this evidence, we inferred that GTP could exert antiapoptotic action through mediating PPAR-*γ*. Interestingly, previous research showed that apoptotic proteins could be attributed to the production of proinflammatory cytokines; meanwhile, increased inflammatory cytokines lead to apoptosis in a positive feedback regulation way [54].

We also studied the cytoskeletal integrity of neurons, by observing the expression of neurofilament protein (NF200) in the spinal cord. NF200 is an essential component of the neuronal and axonal cytoskeleton and is extremely important in the maintenance of neuronal functions following SCI [55]. The degradation of NF200 in axons suggested a certain degree of cytoskeletal breakdown in SCI [35]. This study determined the level of NF200 through quantity analysis. As demonstrated, the level of NF200 in the GTP group was distinctly higher compared with the model group, indicating that GTP treatment had a satisfactory improvement on cytoskeletal integrity. Due to upregulation of PPAR-*γ* facilitating the remodeling of cytoskeletal integrity [56], our results showed a potential association between GTP and cytoskeletal integrity. The result was also consistent with the abovementioned alterations of motoneurons. Meanwhile, the results strongly reinforced the idea that the cytoskeletal integrity of the spinal cord could supply neuronal nutrients [55]. It was also shown that even a small number of surviving axons may bring about dramatic improvement in functional outcomes [57].

Nevertheless, there are a number of limitations to the present study. The present model is based on chronic compression of the spinal cord; however, it was unlikely to fully reflect the progressive nature of human disease. Further research into the mechanisms of GTP on the secondary effect on regeneration following SCI is required.

## 5. Conclusions

**Figure 8:**
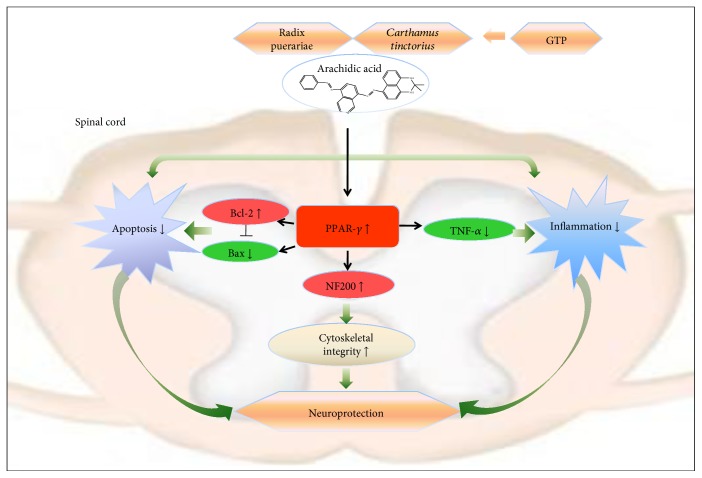
Schematic diagram depicting how GTP modulates the PPAR-*γ* pathway to play a neuroprotection role in CSR. As indicated, arachidic acid is the common component of Radix Puerariae and *Carthamus tinctorius*. By directly activating PPAR-*γ*, GTP could inhibit inflammation and apoptosis by decreasing TNF-*α* and Bax and increasing Bcl-2, respectively. Meanwhile, by aggrandizing NF200, GTP could improve the cytoskeletal integrity.

In conclusion, our experiment pointed to the neuroprotective effect of GTP. We demonstrated that GTP promoted motor function recovery and decreased neuropathic pain after SCI. A protective effect of GTP could be due in part to curtailing inflammation and antiapoptosis while promoting neuron regeneration. The potential mechanisms might be modulated by activating PPAR-*γ* and subsequently inhibiting the expression of TNF-*α* and Bax as well as enhancing the expression of Bcl-2 and NF200 (summarized mechanistic pathways are represented in Figure 8).

## Supplementary Material

Supplementary. Table 1: Database analysis of GTP. Figure 1: Effect of GTP on TNF-α expression in serum. The level of TNF-α in the model group was markedly increased compared to that of the GTP group (P < 0.05). ∗P < 0.05 versus model group.
