# Supplementary material for: Neuroprotective Potential of Gentongping in Rat Model of Cervical Spondylotic Radiculopathy Targeting PPAR-γ Pathway
Source: J Immunol Res. 2017 Nov 5;2017:9152960. doi: 10.1155/2017/9152960 (PMC5694586; doi:10.1155/2017/9152960)
Supplement: Supplementary file 1 — Supplementary. Table 1: Database analysis of GTP. Figure 1: Effect of GTP on TNF-α expression in serum. The level of TNF-α in the model group was markedly increased compared to that of the GTP group (P < 0.05). ∗P < 0.05 versus model group. [file 9152960.f1.pptx]

## Slide 1
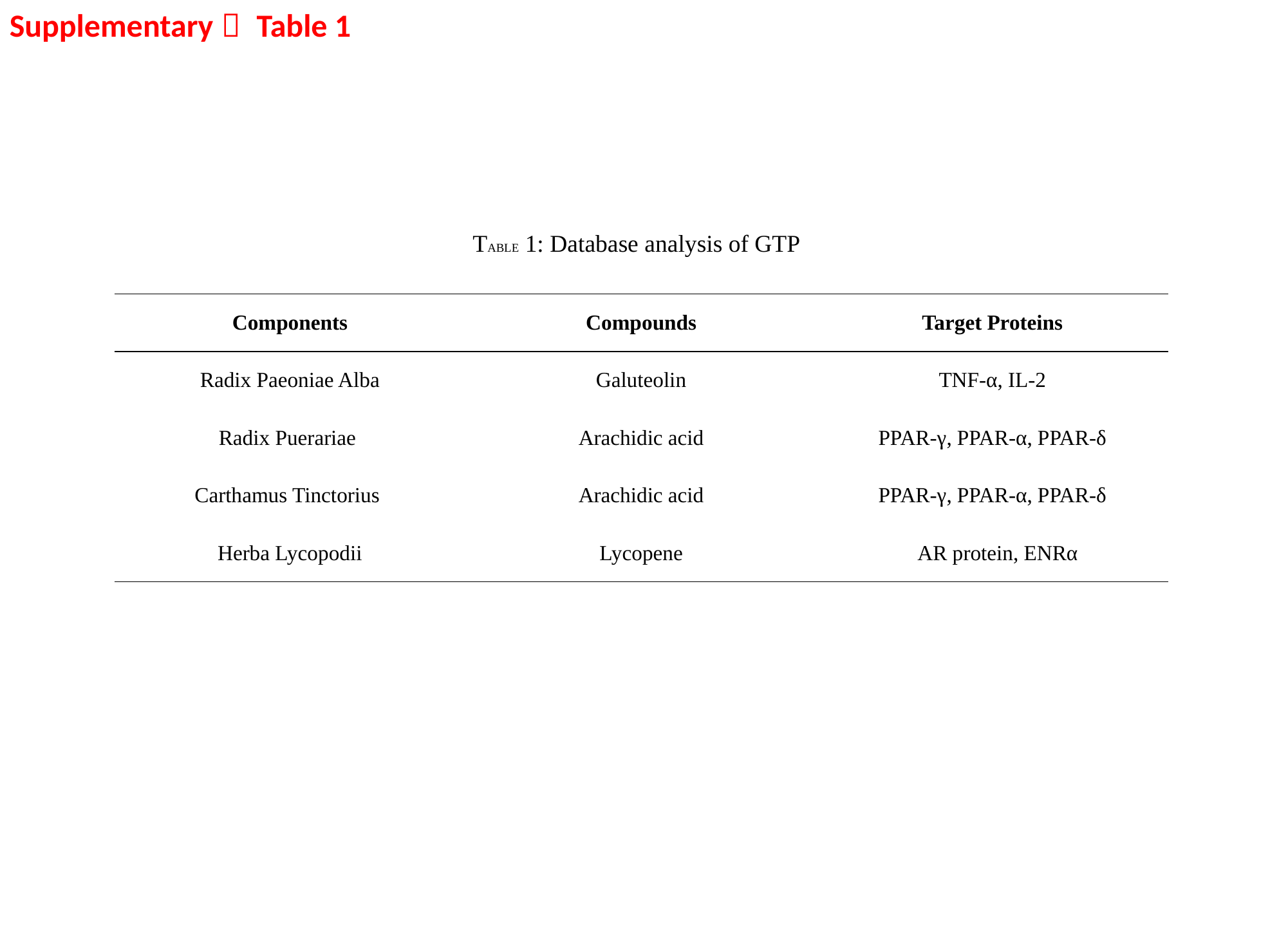

Supplementary： Table 1
| TABLE 1: Database analysis of GTP | | |
| --- | --- | --- |
| Components | Compounds | Target Proteins |
| Radix Paeoniae Alba | Galuteolin | TNF-α, IL-2 |
| Radix Puerariae | Arachidic acid | PPAR-γ, PPAR-α, PPAR-δ |
| Carthamus Tinctorius | Arachidic acid | PPAR-γ, PPAR-α, PPAR-δ |
| Herba Lycopodii | Lycopene | AR protein, ENRα |
