# Supplementary figures and images for: Neuroprotective Potential of Gentongping in Rat Model of Cervical Spondylotic Radiculopathy Targeting PPAR-γ Pathway
Source: J Immunol Res. 2017 Nov 5;2017:9152960. doi: 10.1155/2017/9152960 (PMC5694586; doi:10.1155/2017/9152960)

## Slide 1
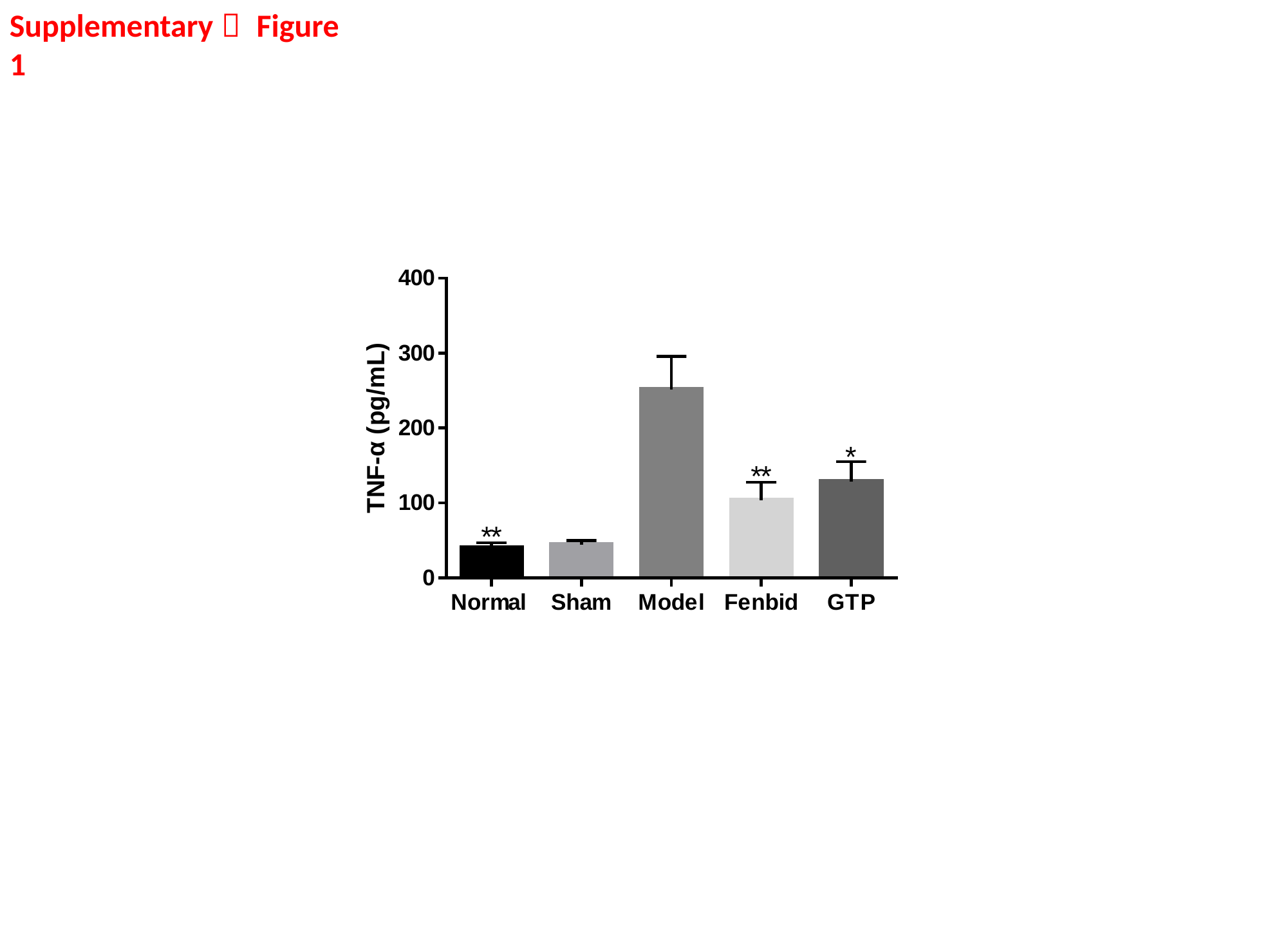

Supplementary： Figure 1

Supplement: Supplementary file 2 [file 9152960.f2.pptx]
